# Gangrene Due to Arterio-Sclerosis1A lecture delivered at Tufts College Medical School, January 11, 1898.

**Published:** 1898-05

**Authors:** Charles Greene Cumston

**Affiliations:** Boston, Mass., Assistant professor of surgical pathology, Tufts College Medical School; Fellow of the American Association of Obstetricians and Gynecologists; corresponding member of the Association of Genito-urinary Surgeons of France; of the Pathological Society of Brussels; of the Electro-therapeutic Society of France, etc.


### BUFFALO MEDICAL JOURNAL.

VOL. XXXVII. MAY, 1898. NO. 10.

### Original Communications.

### GANGRENE DUE TO ARTERIO-SCLEROSIS.[1]

BY CHARLES GREENE CUMSTON, B. M. S., M. D., Boston, Mass.,

Assistant professor of surgical pathology, Tufts College Medical School; Fellow of the American Association of Obstetricians and Gynecologists; corresponding member of the Association of Genito-urinary Surgeons of France; of the Pathological Society of Brussels; of the Electro-therapeutic Society of France, etc.

WHEN the committee on evening lectures kindly asked me to address you, I looked over the ground a little in order that I might select a subject of some practical value and it resulted in the choice of Gangrene produced by arterio-sclerosis. This form of gangrene is a particularly interesting pathological condition for the surgeon as well as for the general practitioner, for it is he who first sees these cases and upon his knowledge of the affection depends the welfare of the patient.

Gangrene from arterio-sclerosis is certainly more frequent in the male, usually occurring between the ages of sixty to seventy and, although extremely rare before forty, it has been observed in younger subjects. The social condition does not appear to have much if any influence, as it is met with in both rich and poor.

The lower limbs are more frequently the seat of the disease and in most cases it begins in the toes. It rarely develops in the hand or arm, although some few cases have been reported. Its appearance on the external ear or end of the nose is very exceptional.

The diseases and systemic intoxications which produce a pathological localisation in the arterial system, such as syphilis, rheumatism, gout, alcoholism, the infectious diseases, malaria and lead poisoning, are looked upon as playing an important part in the etiology of arterio-sclerosis and consequently this type of gangrene. Arterio-sclerosis begins as a chronic endarteritis and later the process of degeneration of the walls of the vessels favors arterial thrombosis, with resulting obliteration of the lumen of the vessel. In some

1. A lecture delivered at Tufts College Medical School, January 11, 1898.

cases, however, no history of any former infection or intoxication can be found, but such patients will usually give a family history of arterio-sclerosis and, as an illustration of this, I will relate a case that occurred in my practice a few years ago:

The patient was a lady of 65 years. Her father died at the age of 69 of dysentery. Her mother died at 55 of heart disease, death being instantaneous. The patient had one sister and two brothers. The sister died of apoplexy at 61 and both brothers died of the same affection in the fifties. Regarding the personal antecedents of the patient, all that is to be noted is that she had scarlet fever at the age of 10 and typhoid at 18. She was always subject to sick headaches. She gave birth to three children at term, five years' interval between each labor, all of which were perfectly normal. There were also two miscarriages, to which no cause could be attributed.

Other than the above-mentioned diseases, and a whooping cough at the age of 25 or 26, the patient had enjoyed excellent health up to September, 1887, when a cerebral embolus occurred, producing a right-sided paralysis, which was entirely recovered from at the end of three months. Again, in 1890, the patient complained of some slight trouble with her sight, and, at the same time, there was a little confusion in the speech, the tongue felt thick and articulation of words was difficult; but all these symptoms cleared up in a few weeks without any other troubles developing.

In the latter part of November, 1894, the patient began to suffer from an intense pain in the heel of the right foot. This pain became very severe and in a few weeks after the onset a small black patch appeared on the great toe, and shortly after this another one on the heel developed. At this time the family physician only applied compresses of some antiseptic solution and, as far as could be learned, nothing else was done. Little by little the gangrene increased and finally involved almost the entire foot. Pain was excessive and the patient was kept from sleeping unless relieved by opiates. By March, 1895, the condition was very bad and there was an elevation of the evening temperature.

I saw the patient for the first time on March 11, 1895, and found the following condition of affairs: The patient was suffering considerably, anorexia was almost complete and the thermometer registered 38.4° C. The right lower limb was in a state of edema, nearly up to the middle of the leg; the toes and the foot up to the malleoli were the seat of a dry gangrene with a very offensive odor. On examination of the arteries I could only detect pulsation at the popliteal space, and consequently it was supposed that the anterior and posterior tibial arteries were entirely obstructed, or nearly so.

With Dr. David W. Cheever, who had also seen the case in consultation, it was decided to remove the limb at the junction of the lower third with the middle third of the thigh. Recovery was uninterrupted and within a few weeks the patient was up and about on crutches. We made an examination of the amputated leg with the following result: The anterior and posterior tibial arteries were extremely thickened and in certain parts not more than a fine wire could be passed through their caliber. The femoral artery, at the point of amputation, presented atheromatous lesions with very thick and pliable chalky patches. The vessel was extremely rigid, although its caliber was not greatly diminished. These vascular lesions were also found in the smaller branches of the arteries of the foot.

Microscopical examination, after decalcification, showed that the walls of the vessels were greatly thinned and their external coat was invaded by a fibrous tissue proliferation. The muscular elements of the walls were much degenerated and the deep veins of the leg were obstructed to a certain extent by large fibrous clots. These clots were made up of red blood corpuscles, united together by a fibrous substance.

We did not see the patient again until November 10, 1896, at which time we were requested to examine the left leg, the following conditions being noted: The left foot was quite dry and the skin on the plantar surface was falling off in scales. The local temperature was sub-normal, being 36.2° C. Over the metatarsal joint, on the inner aspect of the foot, was a patch of hyperemia, the size of a silver dollar, in the center of which was a large suppurating focus covered by a thick dry scab. The same condition was noted on the external aspect of the foot, only the area covered by the hyperemia was a trifle smaller. A suppurating focus also existed in the center of this patch.

Pressure over the tibia gave rise to pitting as far up as the knee, although the size of the limb was in no way increased by the edema. The patient complained of considerable pain, although not intense. Believing that we were dealing with a commencing gangrene, due to the general atheromatous condition of the patient, it appeared possible that the progress of the disease might be perhaps checked by directly acting on the heart and blood-vessels, and the patient was placed on five-drop doses of tincture digitalis twice daily, and a teaspoonful of a 1-100 per cent. solution of nitro-glycerine, to be taken after each meal.

The limb was done up in absorbent cotton, kept in an elevated position, and the suppurating foci washed daily with a solution of creolin, after which they were freely covered with subgallate of bismuth. The result was that after six weeks the condition of affairs was decidedly improved; the scabs had fallen off and the suppurating foci had become completely healed, while the scaly condition of the skin remarked on the sole of the foot had entirely disappeared. The pain it was said vanished after a few days' treatment with the digitalis and nitro-glycerine and the patient was feeling in every way much better. The digitalis and nitro-glycerine were then discontinued and the patient placed on iodide of potassium in fifty centigram doses, twice daily. The iodide treatment was kept up for ten weeks and then the digitalis and nitro-glycerine were again resorted to, although the limb was in an apparently healthy state. From November, 1896, to February, 1897, there was no return of the local trouble, but on the 14th of February the patient died suddenly from what was to all appearances a cerebral hemorrhage.

I have reported this case at length in order to point out how much may be accomplished by medical treatment if instituted at an early date, and also because the case is a typical example of atheromatous gangrene.

Older writers generally divide gangrene into two varieties, the dry and the moist. This classification, which is entirely clinical, is still admitted today, and it is usually the dry form that is met with in atheromatous gangrene. The necrosed part is much smaller in size, but the limb keeps its general shape. The bony projections are made more marked and likewise the depressions. The toes are flexed and dried and it is impossible for the patient to move them. After a time the parts become very hard, tough and resonant on percussion. The color of the affected area becomes darker until it is quite black. The dessication of the tissues is favored by the falling of the epidermis, which produces the formation of blisters in the beginning of the disease. Microscopically, we find a remarkable preservation of the anatomical structures, and we can easily recognise the muscular fibers, the connective and cartilaginous cells, as well as the blood corpuscles.

Now, if we are dealing with a case of moist gangrene, which is extremely infrequent, the tissues are macerated and edematous; their consistency is soft and semi-fluid. The limb has a more or less blackish color, the skin which it covers is easily detached from the underlying tissues, as everything is infiltrated by a serous fluid. Microscopically we can no longer distinguish the various elements of the tissues, but we may find the presence of microörganisms, such as the bacterium thermo and the bacterium catenula, which are the most common organisms found in putrefying tissues.

The condition that is the most striking when we examine the arterial system of a patient with senile gangrene, is the presence of atheromatous foci, ulcerating patches and chalky degeneration. These lesions may be also met with in the large arteries as well as those of small and medium-size caliber, and which produces more or less obliteration. It is rare that the caliber of a vessel is entirely obstructed by an endarteritis and without the help of a thrombus, but the diminution in the caliber that this condition may produce is very considerable.

Forestier performed the autopsy of a man who had died from generalised infection, with a suppurating parotitis following a senile gangrene of the left foot, and he found considerable diminution in the diameter of all the arteries, due to the invasion by a considerable chalky deposit. In other cases which have been reported, in which the artery was entirely obstructed and reduced to the condition of a band of chalk, the interior of the vessel was entirely invaded by the disease.

We do not believe that it is necessary for the caliber of the vessels to be entirely obstructed in order to produce gangrene of the limb. All that is necessary is that there is a decrease sufficiently considerable to prevent the blood from irrigating the limb in sufficient quantity. Now, if a degeneration of the heart muscle be added to the lesion of the vessels, gangrene will certainly occur on account of the loss of force in the cardiac contraction.

Pitres believes that, in the first place, one of the principal arteries of the limb, which later on will be the seat of gangrene, becomes obliterated by a blood or fibrinous clot and then the vessel becomes the seat of an endarteritis. Collateral vessels then develop and it is by them only, or nearly so, that sufficient blood is distributed through the limb to nourish it. There is thus established between the blood carried into the limb and the necessaries of nourishment a kind of equilibrium, which fairly well carries out the nutrition of the limb.

Now, if there is the slightest event which disturbs this equilibrium, a progressive lesion of the principal artery, the flow of blood in the limb will be arrested and gangrene will then occur. Sometimes we meet with circular strictures in arteries of a gangrenous limb, and these strictures act by hindering the circulation in the corresponding extremities and thus diminish the quantity of blood which is necessary to maintain their life. A certain number of strictures will be found along the vessel and part of the blood, which the vessel should allow to pass if its caliber was normal, is forced through at the systole. Arterial tension is consequently lower from this fact. The same phenomena occurs at the point of each stricture, so that we can easily understand what considerable hindrance this form of endarteritis may produce in the circulation, and it is evident that this condition is pre-eminently favorable for the development of gangrene.

In a case reported by Forestier, the arteries of the diseased limbs were perfectly permeable and presented no apparent lesions. The patient was an old woman with a gangrene which had invaded both feet and legs almost symmetrically and which caused her death. The arteries of the lower limbs were found, at autopsy, to be nearly healthy, or at any rate they were not obliterated. On the other hand, the thoracic and abdominal aorta as well as both primary iliacs presented very marked lesions ; they were rough, nearly calcified and transformed into a rigid canal, whose caliber was extremely reduced in size and very irregular, so much so that a pencil could not be inserted into the vessels. In spite of the absence of lesions of the arteries of the limb it would appear that this case was certainly one of gangrene, due to sclerotic changes in the artery and, as a result of this, the vessels were narrowed and only allowed a small quantity of blood to flow through, although too insufficient to keep the lower limbs in good vital condition. Now, when the blood column had gotten through the narrowed vessels and had entered the arteries of normal size its tension was considerably diminished and the result would have been the same as if a ligature had been loosely tied around the femoral vessels, thus allowing only a small quantity of blood to pass through.

The cause of gangrene from arterial sclerosis is consequently entirely due to more or less complete obstruction of the vessel. This obstruction, for that matter, does not depend on a pathological process always of the same nature, and may be due to a proliferation of the endothelium, especially in the small arteries; to chalky degeneration of the walls of the vessels; to a thrombosis; the lining membrane becoming unequal and rough, and thus favoring the deposit of fibrin along the walls; it may also be due to an embolus, a piece of chalky mass becoming detached from the wall in large vessel, as for example, the abdominal aorta, and being carried along until it suddenly obstructs an artery of smaller caliber.

The lesions of the arteries are not the only ones met with, and often the heart is the seat of endocarditis or myocarditis, both of which conditions simply increase the difficulty of the circulation.

Hypertrophy of the heart is also quite frequent, because the organ is obliged to fight against obstructions which prevent the free flow of the blood and is deprived of the action of elasticity of the vessels and consequently the heart becomes hypertrophied.

In a number of cases reported by Pitres and Dumaz the myocardium was extremely soft and yellow in color and in many others the hypertrophy of the organ have been noted. In some cases the mitral valves present marked induration at their base and occasionally the sigmoid valves present lesions of atheroma.

The microscopical lesions of the arteries present nothing of particular note in cases of gangrene and may be summed up briefly as follows: The lesions occur in the endothelial lining, the fibro-elastic layer of the external layer of the vessels, and are intimately bound together on account of the inflammation.

According to Martin, the first lesion occurs in an endarteritis of the vasa-vasorum; the proliferation of the endothelium, by producing more or less obstruction of the caliber of the vessel, prevents the arterial walls from receiving sufficient nourishment, and consequently the nutritive changes take place by imbibition in the external layer, which is the first to be the seat of the disease.

In the arteritis, due to syphilis, Martin considers that the changes are due to the infected blood which acts directly on the endothelium.

Chalky degeneration, which is so very frequent in old men, especially in the arteries of the limbs and brain, commences first, by a chronic endarteritis which commences in the internal layer already hypertrophied, and thus endarteritis is microscopically characterised by a proliferation of the cells in the internal tunic, and by the formation of a corresponding intercellular substance similarly fibrinous in character, which soon gives the membrane a cartilaginous consistency. This cartilaginous tissue may develop regularly in the internal tunic in such a way as to diminish in every sense the caliber of the vessel, or it may develop more especially in certain places and form patches which project into the lumen of the vessel.

Following these lesions, the flow of the blood is slower in the arteries, which are the seat of the disease, and it may go to such an extent that a coagulation of blood takes place. The lesions in the arteries favors and produces the formation of thrombosis and these will have all the same tendency to form in the arteries which have the most active circulation. On account of the projections formed in the lumen of the vessel, the hematoblasts are arrested in their course and fibrin is soon deposited on them and thus a large number of red blood corpuscles are collected in its meshes and a clot is thus formed. When there is an arrest in the circulation the blood coagulates in a mass and we then have a clot, due to stasis. In all cases the permeability of the artery is forever lost.

In 1876 Friedlander described a variety of gangrene, whose symptoms were those of gangrene due to arterio-sclerosis, which differs by its pathological anatomy, having as a characteristic a considerable reduction in the size of the arterial system, and Panas has also reported a case of gangrene due to obliterating endarteritis. Other cases similar to the above have been reported by other observers.

There is between this gangrene by stricture of the arteries, and gangrene due to arterio-sclerosis of the vessels, more than one similar point, and if the arteries are neither hard nor irregular in shape, if there is no tendency to the formation of fibrous tissue, if the chalky degeneration is entirely wanting, and it is often the case in senile gangrene, we are dealing in both cases with gangrene from lesions of the arteries, because the vessels are no longer able to allow a sufficient quantity of blood to pass through them. The clinical symptoms which denote the presence of gangrene in both cases are exactly the same, and it is for this reason that we have mentioned this other form of production of gangrene.

Gangrene from arterio-sclerosis is very often preceded by prodromes, which occur several weeks, or sometimes several months or even a year, before the gangrene of the tissues. Generally pain is the first symptom which occurs and has no particular characteristic which would put the surgeon in the way of making a correct diagnosis. The pain always occurs when there is commencing gangrene from arterial obliteration and is present long before gangrene makes its appearance.

François mentions the case of a patient who noticed pain in his leg when he over-did walking, and this occurred two years before the appearance of gangrene. Pitres mentions the case of a patient eighty-two years old with a gangrene of the foot, and who had suffered for two years previously from pain in the leg. Another case is mentioned by Barth, who had painful symptoms in the right leg four years before the gangrene appeared. In a large number of cases the pain occurred some time beforehand, but in others it is within a few months before the appearance of the local lesions. This pain is generally produced by fatigue, and cold also favors it; later on even moderate walking will produce it and soon it occurs without any cause, even during rest in bed.

The pain is probably due to obliterating arteritis of the vasa nervorum and this neuritis, far from being primary, is probably consecutive to arteritis of the vasa nervorum. Tingling and a heavy feeling in the limbs are also apparent signs of gangrene, as is also the sensation of cold. Examination of the organs of circulation is important, and it will be found that the superficial vessels are hard, knotty, and have a tense feel when the finger is applied to them. Pulsation will be also felt with difficulty or may be absent.

There is hypertrophy of the heart on account of the difficulty of circulation in the vessels and consequently cardiac dulness is increased; soon symptoms appear which can no longer leave any doubt as to the true nature of the disease. At the same time that the pain and heavy sensation increases, the temperature will suddenly fall in a marked manner and absence of pulsation at the root of the diseased limb; in the toes, on the side of the nail or in the interdigital space will be seen a livid plaque of a reddish color, surrounded by disseminated venous circulation. Soon after a blister appears, which contains a fetid serous fluid, and from this time on the phenomena characteristic of gangrene appear. The lowering of the temperature of the diseased limb appears little by little as the decrease of pulsation in the vessels become more and more marked, and although the gangrene has taken place and takes on an essential chronic progress, this lowering of the temperature is very manifest and is easily noticed even by comparing the surface temperature of both legs with the hand. In one case reported the local temperature was 31.2° on the healthy limb and 27.8° for the diseased limb.

Recently another case has been mentioned by Forestier in which he was able to find a difference of more than 2° centigrade between the diseased limb and the healthy one, although in this case it was one of slowly progressing gangrene and at the beginning of the disease. The symptom of painful anesthesia is frequently met with. The patient has intolerable pains in the leg and foot which prevent sleep; nevertheless when pressure is made over the toes he does not feel it and likewise hot poultices when applied to the leg to bring back circulation.

With this loss of sensibility in the part situated below the point of stricture in the artery we may sometimes note a certain degree of hyperesthesia in the region situated above; sometimes the anesthesia is only superficial and when the limb is pricked deeply a dull pain may be complained of by the patient. When the gangrene has extended high up on the foot, or the beginning of the leg, it stops progressing and a line of demarcation occurs between the dead and the living tissues. This line is in the first place shown by a red inflammatory line and soon a process of exudation occurs. The skin becomes ulcerated and a sulcus becomes marked little by little. The lower border of this sulcus is made up of gangrenous tissue, while the other upper border is made up of healthy or nearly healthy tissues because their vitality is extremely poor at this point. Now, this furrow continues to become deeper and deeper, and there is a true absorption of that part of the eschar which is in contact with it and the muscles and aponeuroses and the bone itself is soon divided by this process. This spontaneous amputation takes several months to be completed if an operation is not performed.

The local phenomena which have just been described are not the only ones which occur in this form of gangrene. A large part should be given to the general symptoms because they are frequent and of considerable gravity. The pain is often sufficiently sharp to produce a restlessness, insomnia, and frequently there is delirium with fever; the patient presents extreme prostration, there is anorexia, a profuse diarrhea occurs, fever declares itself and the patient is quickly carried off by a true septicemic process. All these symptoms are due without doubt to the absorption into the economy of a part of the tissues and liquids from the focus of the gangrene and the autotoxemia is all the more serious because it occurs in elderly people having a generalised arterial sclerosis, and consequently whose kidneys are insufficient and whose organism no longer can defend itself sufficiently against toxemia or the invading microörganisms; consequently it may be said that the patient can die of his gangrene without any other complication occurring.

Senile gangrene usually follows a slow and chronic progress, especially when we are dealing with the dry form. The local points of gangrene remain for several days or months without progressing. They become limited in the first place on one toe and then later on invade another, and thus little by little they appear over the entire hand, foot or leg, or the fore-arm, elbow or the knee. At other times the progress is more rapid, and the gangrene occurs at once, the patient suffering only one or two days, and at the same time the temperature becomes lower, movement is very painful, the leg takes on a bluish livid color, the epidermis becomes raised up as in a putrefied limb; in other words, a rapid type of gangrene has declared itself, which by its manifestations is very similar to a gangrene due to embolus.

When we are dealing with a case of gangrene in a patient in which no other local manifestation has as yet occurred, the diagnosis is extremely difficult and often impossible to establish. In many cases rheumatism has been thought to be present, on account of the pain, and especially when this is localised over a joint. When the pain takes on a shooting type of burning and heat, it may be taken for a symptom of a lesion of the spinal cord. In the same way the symptoms of paralysis, which have been noted in certain cases, and which present their greatest intensity immediately in the beginning, have also been taken for paralysis of central origin.

When the gangrene has become marked, and it has become manifest by the local lesions, the diagnosis can be made. No one would mistake a patch of gangrene for ecchymosis, and it would also not be taken for a periarthritic ulcer, if it commences over the big toe, the border of the foot or the heel. This is only mentioned in order to prevent any error occurring. Now, when the diagnosis of gangrene is made, the next consideration is to know with what type we are dealing. If it is one following directly a wound or a contusion, burn or freezing of the limb, the patient will give us the necessary information on this subject. If it is a gangrene due to some internal cause its nature becomes far more difficult and delicate to determine. Inquiries of the patient regarding the possible history of infection should always be made in the first place and the heart should be carefully examined. The urine must be examined to ascertain if it contain albumen and especially sugar, which, if it is present, will allow us to make a diagnosis of diabetic gangrene. The sudden appearance of this affection, announced by pain, sudden lowering of the temperature and ischemia of the limb would naturally point to an embolus as a cause.

The diagnosis of gangrene due to a generalised atheroma is very difficult. It is true that in this case the pain is, in most instances, less severe, but what will lead us to suppose that we are dealing with this form of gangrene is when the pulsation of the arteries can no longer be found, and, also, the vessel itself will escape being felt by the exploring fingers. Clinically, it is often very difficult to make an exact diagnosis, because the edema will often prevent direct examination of the arteries of the diseased limb, and in this case an examination of the superficial vessels must be made, such as the temporal, in order to ascertain their condition.

The age of the patient has also a certain importance as regards the diagnosis, but it must be remembered that gangrene, due to an obliterating endarteritis, often develops in people who are not advanced in age. Now, we will suppose that our diagnosis is made and that gangrene has commenced to make its appearance. What should be the measures of treatment? Should we delay operation or, on the contrary, would it be better to interfere immediately? If we are dealing with a well-limited gangrene and if the neighboring tissues are in a normal condition; if the arteries are still sufficiently elastic, so that their pulsation can be felt; if on the other hand the patient is still strong, with a heart in good condition without myocarditis; if nothing abnormal is found in the urine—in this case no hesitation is permitted, but we must operate.

An operation is to be more fully considered if, at a certain time,—for example, when elimination takes place,—suppuration with symptoms of infection occur. But if the gangrene takes on a serpiginous character, and if gangrenous points appear in the midst of healthy tissues, and if the pathological process has any tendency to become limited, if the pulsation of the arteries cannot be felt, and if the patient is in a cachectic condition, with a heart on the point of failing, and the kidneys doing their work in a defective way—in this case we must abstain from operating. We should be reserved in the prognosis and wait a while before making up our minds if it is still impossible to foresee, even approximately, at what point the gangrene will stop, if the limb presents an ecchymotic aspect, if it is markedly colder than its fellow limb, as well as if it is painful and useless. The prognosis of atheromatous gangrene is always extremely serious, because it often produces very extensive mutilation of the limb, frequently relapses, and is often in itself a cause of death, as we have already pointed out.

We must also bear in mind that complications may arise which hasten the progress of events. These complications are of two kinds: The first are due to the poisoning of the organism, and is a direct consequence of the gangrene. The other complications are due to arterio-sclerosis, and are only manifestations of this degeneration, as is the gangrene itself.

Septicemia is the most dangerous as well as the most frequent complication and as an illustration of this I would mention a case reported by Mendel of a man of sixty-seven years, presenting a senile gangrene. The patient died of septicemia in a few days and the obliterating phlebitis in the tibial vein, the fluid condition of the blood, as well as the great softness of the spleen, left no doubt as to the anatomical diagnosis.

We shall not say anything regarding the lesions presented in the arteries in cases of advanced atheroma, but I would mention the fact that in these cases of septicemia the liver and spleen show signs of an infectious process. In other cases, instead of a general infection occurring at once, a senile gangrene may produce a lymphangitis and abscesses which develop in healthy tissue some distance from the gangrene itself. Pneumonia and broncho pneumonia have also been mentioned as complications, as well as phlebitis and embolus. Other complications that may be mentioned are nephritis, myocarditis and cerebral hemorrhage.

Regarding the treatment there is little to be said, but the first important and one of the essential conditions of success is to keep the patient's strength, and for this the free administration of quinine is of first importance; as is well known, this drug is both a tonic and an antiseptic, and its action in these cases is quite manifest.

In cases where there is a tendency to gangrene, as in the case taken as the text of this lecture, it may be accomplished by medical treatment. In the first place the judicious use of nitro-glycerine, combined with an infusion of digitalis, may act like a charm and prevent gangrene from occurring for at least a certain length of time. The diet should be nutritious and easily digested and the patient should be kept as quiet as possible. Besides this the suspected limb should be maintained elevated in a horizontal position and enveloped in a thick layer of absorbent cotton in order to maintain a suitable temperature. Amputation is indicated when the gangrene is pronounced, but on the condition that the abdominal organs, heart and lungs are in good condition, as well as the general strength of the patient.

To sum up we may say that there are three manners of interfering. In the first place we have what we call "tardy interference," which is simply a section of the bone and evening off of the stump after the gangrene has ceased to progress and the line of demarcation has become marked. We call it a "secondary operation" one in which the demarcation has become distinct, and then we amputate high up above the lesion, while an immediate operation is that one which is performed as soon as the symptoms of gangrene have made their appearance and are certain.

It would be impossible to discuss fully the proper treatment of atheromatous gangrene, because each case is a law unto itself, and we can only be governed by the actual conditions presented in a given case, when we should take into consideration the various pathological and symptomatic phenomena present and act accordingly.

871 Beacon Street.

